# NRPS and PKS pathways in actinomycetes: A review of biosynthetic diversity and bioactive potential

**DOI:** 10.1016/j.crphar.2026.100254

**Published:** 2026-04-20

**Authors:** Abhishek Chauhan, Manisha Gurnani, Jayati Arora, Ritu Chauhan, Anuj Ranjan, Naveen Chandra Joshi, Laurent Dufossé, Tanu Jindal

**Affiliations:** a Amity Institute of Environment Toxicology and Safety Management, Amity University, Noida, U.P, India; b Centre for Global Health Research, Saveetha Medical College and Hospitals, Saveetha Institute of Medical and Technical Sciences, Thandalam, Chennai, 602105, India; c Department of Biotechnology, Graphic Era Deemed to be University, Dehradun, Uttarakhand, 248002, India; d Amity Institute of Microbial Technology, Amity University, Noida, 201313, India; e Chemistry and Biotechnology of Natural Products, CHEMBIOPRO, Université de La Réunion, ESIROI Agroalimentaire, 15 Avenue René Cassin, CS 92003, CEDEX 9, Saint-Denis, F-97744, France

**Keywords:** Biosynthesis, Secondary metabolites, Drug discovery, Non-ribosomal peptide synthase, Polyketide synthase

## Abstract

Actinomycetes are Gram-positive bacteria renowned for their capacity to produce chemically diverse secondary metabolites with significant pharmaceutical relevance. Widely distributed across marine and terrestrial environments, they exhibit remarkable metabolic adaptability that supports the synthesis of novel compounds under environmental stress. This review examines the biosynthetic diversity of actinomycetes, with a focus on non-ribosomal peptide synthetases (NRPSs) and polyketide synthases (PKSs), the principal enzymatic systems responsible for complex metabolite assembly. A clear distinction is made between synthetases (ATP-dependent enzymes such as NRPSs) and synthases (ATP-independent enzymes such as PKSs), addressing a key conceptual ambiguity. The modular architecture of NRPSs, including adenylation, thiolation, and condensation domains, is discussed in relation to peptide biosynthesis, while PKS systems are outlined based on their classification (Types I–III), domain organization, and chain elongation mechanisms. Hybrid PKS–NRPS pathways and inter-domain interactions are further highlighted for their role in expanding chemical diversity. Collectively, these biosynthetic systems highlight the potential of actinomycetes as a rich source of therapeutically relevant compounds, particularly in antimicrobial and anticancer drug discovery.

## Highlights

• Actinomycetes produce diverse NRPS- and PKS-derived metabolites with major pharmaceutical value.
• NRPS and PKS enzymes use modular domains to build complex bioactive molecules.
• Marine actinomycetes generate unique metabolites due to their adaptation to extreme environments.
• Hybrid PKS–NRPS pathways create highly potent antimicrobial and anticancer compounds.
• Genome mining shows actinomycetes contain many unexplored biosynthetic gene clusters for new drugs.

## Graphical abstract

**Figure:**
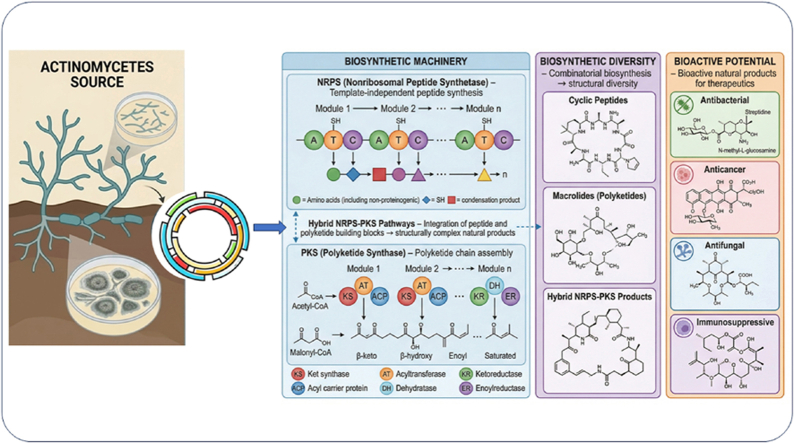

## 1 Introduction

Actinomycetes are a diverse group of filamentous, Gram-positive bacteria characterized by a complex life cycle and high G + C content. They belong to the phylum Actinobacteria and represent one of the largest taxonomic groups within the domain Bacteria (Sharma et al., 2019). Their filamentous morphology, resembling fungal hyphae, facilitates efficient substrate colonization and nutrient acquisition under diverse environmental conditions (Mondal and Thomas, 2022).

These microorganisms are widely distributed across terrestrial and aquatic ecosystems, including soil, marine sediments, mangrove environments, and plant-associated habitats, where they tolerate extreme conditions such as variations in temperature, pH, salinity, and nutrient availability (Mondal and Thomas, 2022; Jagannathan et al., 2021). This adaptability contributes to their ecological significance and metabolic versatility. Actinomycetes also play a key role in the degradation and recycling of organic matter in different environments (Mondal and Thomas, 2022). Mangrove ecosystems, in particular, have been recognized as promising reservoirs of metabolite-producing actinomycetes, with isolates harboring NRPS and PKS biosynthetic gene clusters and exhibiting antibacterial activity against pathogens such as *Escherichia coli*, *Staphylococcus aureus*, and *Listeria monocytogenes* (Setyati et al., 2021).

A defining characteristic of actinomycetes is their ability to produce chemically diverse secondary metabolites, including alkaloids, terpenoids, flavonoids, and antibiotics (Yi et al., 2025). These metabolites exhibit a broad spectrum of biological activities and have been widely applied in pharmaceutical and biotechnological fields (Kim, 2021a, 2021b; Al-Shaibani et al., 2021). Actinomycetes, particularly the genus *Streptomyces*, are a major source of clinically important antibiotics and account for a substantial proportion of known natural products (Al-Shaibani et al., 2021; De Simeis and Serra, 2021). Advances in genome mining and synthetic biology have further enhanced the exploration and engineering of their biosynthetic potential (Salwan and Sharma, 2020).

The biosynthesis of these metabolites is governed by biosynthetic gene clusters (BGCs), among which NRPSs and PKSs represent key enzymatic systems (Komaki et al., 2018). Despite extensive research, only a small fraction of actinomycetes has been cultured, indicating a vast unexplored reservoir of metabolic potential (Hofer, 2018). Advances in genome mining and metagenomics have revealed numerous cryptic gene clusters, providing new opportunities for natural product discovery (Russell and Truman, 2020). Together, these approaches enable the identification of novel metabolites synthesized by NRPS and PKS systems and facilitate access to previously untapped microbial resources (Nikolouli and Mossialos, 2012; Arulprakasam and Dharumadurai, 2021). Integrated genomics and metabolomics have further strengthened the discovery pipeline, particularly in marine-derived *Streptomyces* (Paulus et al., 2017). Rare actinobacteria also represent an underexplored reservoir of metabolites, and advances in molecular tools have enabled efficient identification and engineering of their biosynthetic pathways (Amin et al., 2020). Marine actinomycetes, in particular, are gaining attention as valuable sources of new compounds with therapeutic potential, including antileishmanial agents (Nair and Abraham, 2020; Davies-Bolorunduro et al., 2021).

A systematic understanding of NRPS and PKS pathways is therefore essential to harness the chemical diversity of actinomycetes and support future therapeutic development.

## 2 Physiological aspects of adaptation strategies

Actinomycetes exhibit diverse growth characteristics and physiological adaptability, enabling their survival under varying environmental conditions. Studies have shown that actinomycete isolates can grow across a range of NaCl concentrations (1–7%), with limited tolerance at higher concentrations (10–20%), and display optimal growth temperatures between 28 and 30 °C, although some species can tolerate temperatures up to 40 °C (Mondal and Thomas, 2022). Actinomycetes exhibit great diversity in various characteristics, including moisture tolerance, habitat, optimal pH, and thermophilicity. They also exhibit variability in moisture tolerance, pH preference, and thermophilic or acidophilic/alkaliphilic nature, reflecting their ecological versatility (Jagannathan et al., 2021).

Two Gram-positive and aerobic actinomycetes, strains SCSIO 64649T and SCSIO 03032, were identified in a separate study. Morphological diversity among actinomycetes is characterized by the formation of branched substrate mycelia and aerial hyphae, which may differentiate into spore chains with distinct structural features. For instance, strains SCSIO 64649T and SCSIO 03032 demonstrated well-developed aerial hyphae and spiral spore chains with smooth-surfaced spores, along with differential growth on various culture media such as ISP and nutrient agar (Shi et al., 2022).

Another study evaluated the morphology of several Actinomycete isolates. Variations in growth patterns and sporulation have also been reported among different isolates. Certain strains exhibit rapid surface growth without spore formation, while others display *Streptomyces*-like morphology with well-developed aerial hyphae and delayed growth, often penetrating the agar surface during colony development (Shirling and Gottlieb, 1966; Flärdh and Buttner, 2009).

Marine Actinomycetes have adapted to various extreme living conditions ranging from high pressures (with a maximum of 1100 atm) and anaerobic conditions at temperatures just below 0 °C on the deep-sea floor to high acidic conditions (pH as low as 2.8) at temperatures of over 100 °C near hydrothermal vents at the mid-ocean ridges (Manivasagan et al., 2013). Marine actinomycetes demonstrate remarkable adaptation to extreme environmental conditions, including high hydrostatic pressure, low temperature, limited nutrient availability, and reduced light penetration in deep-sea habitats (Manivasagan et al., 2013). In addition, coral-associated marine actinomycetes have been identified as promising sources of novel bioactive metabolites with antibacterial, antifungal, and cytotoxic activities, highlighting these ecosystems as largely untapped reservoirs for drug discovery (Siro et al., 2022). These organisms have different characteristics from those of terrestrial counterparts and therefore might produce different types of bioactive compounds (Mahmoud and Kalendar, 2016) . The deep-sea environments are bathyal zone from depths below 2,000m, the abyssal zone from depths ranging between 2,000m and 6,000m, and the hadal zone from depths below 6,000m. These environments, categorized into bathyal, abyssal, and hadal zones, require organisms to undergo significant biochemical and physiological adjustments to sustain growth and metabolic activity (Kamjam et al., 2017). Subterranean environments such as caves also represent underexplored ecological niches harboring rare actinomycetes with diverse biosynthetic gene clusters, including NRPS and PKS pathways, highlighting their potential for novel antimicrobial discovery (Gonzalez-Pimentel et al., 2022). Deep-sea species must adjust their biochemical processes to survive in low temperatures and exponentially decreased light intensity with depth in the water column. According to Haefner (2003), in cold deep-sea mud, the diversity of life can be remarkably high, with species richness rivaling that of tropical rainforest (Kamjam et al., 2017). Such adaptations contribute to the production of structurally distinct bioactive compounds, often differing from those produced by terrestrial actinomycetes, thereby enhancing their potential in drug discovery (Okoye et al., 2020). Marine rare actinomycetes further represent an underexplored reservoir of structurally diverse and novel bioactive compounds with significant pharmacological potential (Subramani and Sipkema, 2019). The high microbial diversity observed in deep-sea sediments further highlights the ecological and biotechnological importance of marine actinomycetes (Kamjam et al., 2017).

### 2.1 Insights into non-ribosomal peptide synthase and polyketide synthase pathways

Actinomycetes are known to encode various BGCs for secondary metabolites in their genomes, with NRPS and PKS pathways being associated with about half to three quarters of the clusters. This indicates that non-ribosomal peptides, polyketides, and their hybrid compounds constitute the major classes of secondary metabolites in actinomycetes, many of which exhibit significant pharmaceutical applications including antibiotics, anticancer agents, and immunosuppressants (Komaki et al., 2018). Interestingly, some deep-sea samples have shown higher productivity of actinomycetes isolation using methods such as no heat pre-treatment, dry and stamp method, and low temperature incubation (Kamjam et al., 2017).

On average, each strain of actinomycetes has 30–40 gene clusters involved in secondary metabolic biosynthesis, the majority of which are obscure. For instance, the genus *Streptomyces*, a major producer of bioactive compounds, possesses approximately 25–70 BGCs, many of which remain silent under standard laboratory conditions. Doroghazi et al. studied the genomes of six actinomycetes extensively and reported that some genera have a greater percentage of NRPS or PKS natural products compared to other genera. Additionally, Hifnawy et al. highlighted the immense potential of the actinobacteria phylum for natural product research, with the genus *Micromonospora* being a model system for this purpose. This genus contains a large number of BGCs (2387 clusters grouped into 1033 families), predominantly belonging to type I PKS and NRPS pathways, indicating extensive biosynthetic diversity (Siro and Donald, 2023).

To increase the chances of yielding novel antibiotics, strains belonging to novel taxa must be able to produce secondary metabolites, possess diversified pathways for secondary metabolism, present significant genetic diversity, be retrievable in large numbers, and be amenable to scale-up for large-volume fermentation (Newman and Cragg, 2016; Demain and Sanchez, 2009). Biosynthetic gene clusters encoding NRPS and PKS systems are therefore considered prime targets for natural product discovery due to their ability to generate structurally diverse and pharmacologically active compounds (Siro and Donald, 2023).

NRPS use C–N bonds to incorporate amino acids, on the other hand PKS catalyzes the formation of C–C bonds by the addition of carboxylic acid in monomeric form. NRPSs function as ATP-dependent synthetases that incorporate amino acid substrates, whereas PKSs act as synthases that catalyze carbon–carbon bond formation using acyl-CoA precursors. Both PKSs and NRPSs are huge enzymes that are organized into modules based on their catalytic pathways. Each module typically contains a thiolation (T) domain carrying intermediates via a phosphopantetheinyl (PPT) arm derived from coenzyme A, ensuring efficient substrate transfer between catalytic domains.

**Fig. 1:**
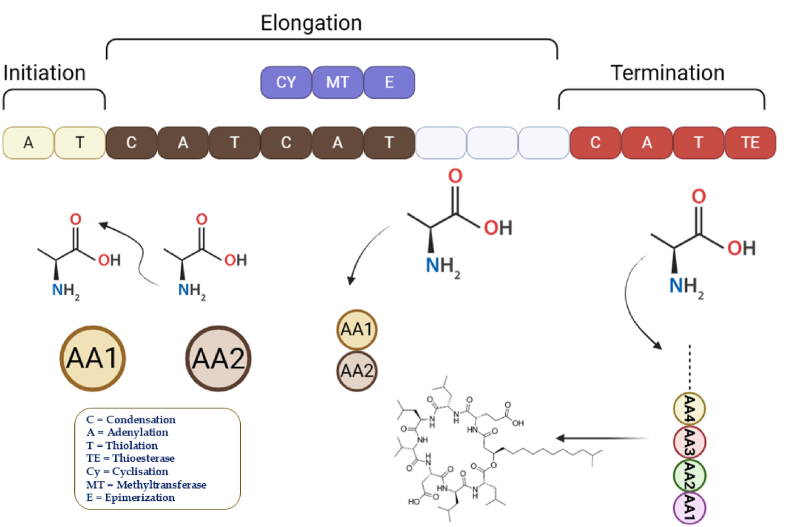
Overview of NRPS biosynthetic pathways in actinomycetes, showing their modular domain organization and roles in the synthesis of diverse bioactive compounds.

The past ten years have seen remarkable advancements in genome sequencing, which has led to an increase in the number of genes known to code for modular PKSs and NRPSs. Sequence analysis has revealed extensive diversification of PKS and NRPS systems through domain rearrangements, module duplication, and evolutionary modifications, contributing to the structural complexity of natural products (Pang et al., 2016). The modular architecture and biosynthetic mechanisms of NRPS and PKS systems are illustrated in Fig. 1.

### 2.2 Non-ribosomal peptide synthetases (NRPSs): structure and function

Non-ribosomal peptide synthetases (NRPSs) are large multi-modular enzyme complexes responsible for the biosynthesis of non-ribosomal peptides (NRPs), a diverse group of secondary metabolites with significant biological activities. These peptides are synthesized independently of the ribosomal machinery and incorporate a wide range of substrates, including proteinogenic and non-proteinogenic amino acids, fatty acids, and heterocyclic compounds, contributing to their structural diversity (Iacovelli et al., 2021).

NRPSs function as ATP-dependent synthetases organized in a modular assembly-line fashion, where each module is responsible for the incorporation of a specific substrate into the growing peptide chain. These large, multimodular enzyme complexes catalyze ribosome-independent peptide synthesis through highly regio- and stereospecific reactions, with catalytic domains coordinating substrate selection, activation, modification, and chain elongation to ensure precise product formation (Strieker et al., 2010). Their size ranges from simple single-module enzymes (∼100 kDa) to large multi-enzyme complexes exceeding 1.8 MDa (Iacovelli et al., 2021).

#### 2.2.1 Biosynthesis of non-ribosomal peptides

NRP biosynthesis occurs through a thiotemplate mechanism in which intermediates remain covalently bound to the enzyme complex during chain elongation**.** The process is initiated by the adenylation (A) domain, followed by substrate transfer to the peptidyl carrier protein (PCP/T domain), and subsequent peptide bond formation catalyzed by the condensation (C) domain (Khabthani et al., 2021). The final peptide product is typically released by a thioesterase (TE) domain through hydrolysis or cyclization, while in some cases reductase domains may also facilitate product release (Walsh, 2004). NRPs exhibit a wide range of structural modifications, including methylation, glycosylation, and incorporation of D-amino acids, resulting in diverse biological activities such as antimicrobial, anticancer, and immunosuppressive properties (Agrawal et al., 2017). Despite their therapeutic importance, the complexity of NRPS biosynthetic systems poses challenges for engineering; however, recent advances in synthetic biology have enabled the emulation of NRPS-derived peptides using ribosomal pathways, facilitating the design of novel bioactive compounds (Mordhorst et al., 2023).

#### 2.2.2 Modular organization and domain architecture

The functional organization of NRPSs is based on repeating modules, each containing essential catalytic domains responsible for peptide elongation. A minimal NRPS module consists of three core domains: adenylation (A), thiolation (T/PCP), and condensation (C) domains (Felnagle et al., 2008). The adenylation domain selects and activates specific substrates in an ATP-dependent manner, forming aminoacyl-AMP intermediates. The thiolation domain carries the activated substrate via a phosphopantetheinyl arm, while the condensation domain catalyzes peptide bond formation between adjacent substrates (Challis and Naismith, 2004). Additional domains such as epimerization, methyltransferase, heterocyclase, and reductase may be present, contributing to further structural diversification of NRPs.

##### 2.2.2.1 Adenylation (A) domain

The adenylation domain acts as a “gatekeeper” by determining substrate specificity and catalyzing ATP-dependent activation of amino acids. It consists of two subdomains forming a substrate-binding pocket and plays a key role in defining the final peptide structure (Marahiel, 2009a, 2009b; Iacovelli et al., 2021).

##### 2.2.2.2 Thiolation (T/PCP) domain

The thiolation domain serves as a carrier unit that binds activated substrates via a phosphopantetheinyl arm and facilitates their transfer between catalytic domains**.** It interacts with multiple domains in a coordinated manner during peptide synthesis (Felnagle et al., 2008).

##### 2.2.2.3 Condensation (C) domain

The condensation domain catalyzes peptide bond formation through nucleophilic attack between adjacent aminoacyl intermediates, ensuring directional chain elongation (Finking and Marahiel, 2004).

##### 2.2.2.4 Thioesterase (TE) domain

The thioesterase domain is responsible for product release through hydrolysis or cyclization and plays a critical role in determining the final structure of NRPs (Felnagle et al., 2008).

#### 2.2.3 Types of NRPS systems

Based on biosynthetic organization, NRPSs are classified into linear (Type A), iterative (Type B), and nonlinear (Type C) systems, each contributing to distinct structural outcomes (Iacovelli et al., 2021).

Linear NRPSs follow a colinear relationship between modules and peptide sequence.

Iterative NRPSs reuse modules for repeated substrate incorporation.

Nonlinear NRPSs involve domain-level reuse, enhancing structural complexity.

#### 2.2.4 Stand-alone NRPS systems

Certain NRPS gene clusters lack typical modular organization and consist of stand-alone domains or didomain proteins that function collaboratively in peptide biosynthesis. These systems often activate unusual substrates and expand metabolic diversity (Iacovelli et al., 2021).

### 2.3 Polyketide synthases (PKSs): classification and mechanism

Polyketide synthases (PKSs) are multifunctional enzyme systems responsible for the biosynthesis of structurally diverse polyketide compounds in actinomycetes. These metabolites include antibiotics, antifungals, antitumor agents, anthelmintics, and immunosuppressants, highlighting their significant pharmaceutical importance. Actinomycetes often harbor genes encoding multiple biosynthetic systems, including PKS-I, PKS-II, and NRPS, within a single strain.

#### 2.3.1 Classification of PKS systems

PKSs are broadly classified into three types—Type I, Type II, and Type III—based on their structural organization and catalytic mechanisms.

**Type I PKSs** are large, multifunctional modular enzymes commonly found in bacteria and fungi. These systems operate either as **modular (assembly-line) PKSs**, where each module catalyzes a single elongation step, or as **iterative PKSs**, where the same domains are reused for multiple cycles (Nivina et al., 2019a, 2019b).

**Type II PKSs** consist of discrete, monofunctional enzymes that act iteratively and are primarily involved in the synthesis of aromatic polyketides in bacteria.

**Type III PKSs** are relatively simple homodimeric enzymes, mainly found in plants and some microorganisms, which catalyze polyketide formation without the requirement of.

#### 2.3.2 Domain organization and catalytic mechanism

A typical Type I PKS module contains three core domains: acyltransferase (AT), acyl carrier protein (ACP), and ketosynthase (KS), which together mediate substrate selection, transfer, and chain elongation through decarboxylative condensation reactions.

**Fig. 2:**
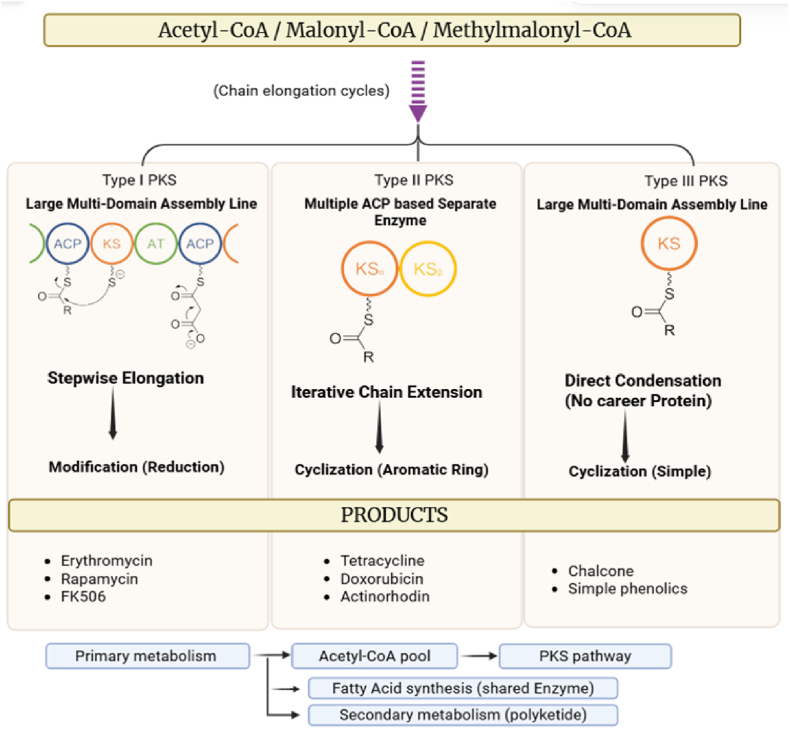
Schematic representation of polyketide synthase (PKS) biosynthetic pathways showing domain organization and polyketide chain elongation.

The ACP domain carries the growing polyketide chain via a phosphopantetheine arm, enabling efficient substrate transfer between catalytic sites. Additional modifying domains such as ketoreductase (KR), dehydratase (DH), enoyl reductase (ER), and methyltransferase further contribute to structural diversification. The completed polyketide chain is typically released by a thioesterase (TE) domain, followed by post-synthetic tailoring reactions such as oxidation, cyclization, and group transfer, resulting in structurally complex metabolites (Zhang et al., 2017a, 2017b, 2017c, 2017d). The domain organization and biosynthetic mechanism of PKS systems are illustrated in Fig. 2.

#### 2.3.3 Biosynthesis of aromatic polyketides (Type II PKS)

Type II PKS-mediated biosynthesis begins with the loading of acetate-derived precursors onto ACP, followed by iterative chain elongation using malonyl-CoA extender units**.** This process leads to the formation of a poly-β-keto chain, which is subsequently modified by enzymes such as ketoreductases and aromatases to generate aromatic polyketide cores (Zhang et al., 2017a, 2017b, 2017c, 2017d). The resulting metabolites include important classes such as anthracyclines, tetracyclines, and angucyclines, which exhibit significant therapeutic activities (Hertweck et al., 2007a, 2007b).

#### 2.3.4 Type III PKS and simplified catalytic systems

Type III PKSs function as homodimeric enzymes that catalyze iterative condensation reactions using acyl-CoA substrates, leading to the formation of linear polyketide intermediates followed by cyclization (Shimizu et al., 2017)**.** These systems are structurally simpler but contribute to diverse metabolite production.

#### 2.3.5 Functional diversity and biosynthetic significance

The structural diversity of polyketides arises from variations in extender units, domain composition, and reduction patterns, enabling PKS pathways to generate compounds occupying unique chemical space not accessible through other biosynthetic routes (Cai and Zhang, 2018). Both PKS and NRPS systems exhibit modular organization and may include additional tailoring domains such as methyltransferases, epimerases, and reductases, which enhance chemical diversity (Dhakal et al., 2019).

### 2.4 Hybrid PKS–NRPS synthases

Hybrid synthases are enzymes that combine functional core domains of both PKSs and NRPSs. These systems integrate the biosynthetic logic of both pathways, enabling the assembly of structurally complex and pharmacologically important natural products**.** These enzymes use a common thiotemplate mechanism for loading and transporting substrates, with NRPSs utilizing PCP domains and PKSs utilizing ACP domains. This coordinated mechanism facilitates seamless transfer of intermediates between modules, resulting in the formation of hybrid metabolites with enhanced chemical diversity.

**Fig. 3:**
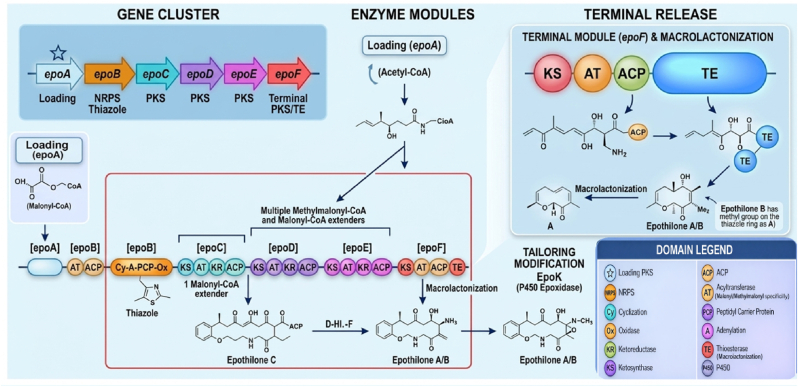
Organization of the epothilone biosynthetic gene cluster (epoA–epoF), corresponding PKS/NRPS enzyme modules, and terminal macrolactonization steps illustrating domain architecture (KS, AT, ACP, KR, DH, ER, TE), stepwise chain elongation, intermediate formation, and conversion of epothilone C/D to epothilone A and B.

Examples of PKS-NRPS hybrid products include the anticancer agents bleomycin and epothilone, the antibacterial and antifungal paenilamicins, and the antibiotic zwittermicin A. The biosynthetic organization of a representative hybrid PKS–NRPS system, such as epothilone, is illustrated in Fig. 3.

#### 2.4.1 Organization and types of hybrid systems

PKS-NRPS hybrids can either be organized in the same polypeptide chain (tethered type) or in separate subunits, where standalone enzymes of one kind are coupled with modular systems of the other (non-tethered type). In tethered systems, PKS and NRPS modules are arranged sequentially within a single polypeptide, allowing direct substrate channeling between domains. In contrast, non-tethered systems involve independent enzyme complexes that interact through specific docking domains to facilitate intermediate transfer (Iacovelli et al., 2021).

#### 2.4.2 Inter-domain communication and functional coordination

In both cases, effective communication is essential to coordinate the transport of substrates and intermediates across the hybrid system. Efficient inter-domain communication ensures proper timing and fidelity of biosynthetic steps, which is critical for the successful assembly of hybrid molecules**.** In tethered type hybrids, specific linker regions play a crucial role in these interactions, while non-tethered type hybrids require special docking domains.

#### 2.4.3 Biosynthetic and biotechnological significance

The ability of hybrid PKS–NRPS systems to combine diverse enzymatic functionalities enables the production of molecules with unique structural features and enhanced biological activities**.** Due to their communication capabilities, there is significant interest in engineering hybrid PKS-NRPS systems for the production of novel compounds (Iacovelli et al., 2021).

## 3 Bioactive compounds from actinomycetes via NRPS, PKS, and hybrid pathways

Actinomycetes are a rich and well-established source of structurally diverse and pharmacologically important secondary metabolites, primarily synthesized through non-ribosomal peptide synthetase (NRPS), polyketide synthase (PKS), and hybrid PKS–NRPS pathways. These complex biosynthetic systems enable the generation of a wide array of bioactive compounds with significant applications in pharmaceuticals, particularly as antibiotics, anticancer agents, and immunosuppressants (Komaki et al., 2018; Nivina et al., 2019a, 2019b; Pang et al., 2016). Details of bioactive compounds produced by actinomycetes through NRPS, PKS, and hybrid pathways are summarized in Table 1. Polyketides represent a dominant class of metabolites, with numerous compounds exhibiting potent biological activities. For example, Streptomyces species derived from marine environments produce compounds such as sharkquinone and resistomycin, which demonstrate cytotoxic effects against multiple cancer cell lines. Additionally, aromatic polyketides such as angucyclines and anthraquinones exhibit strong antimicrobial activity, particularly against methicillin-resistant *Staphylococcus aureus* (MRSA), emphasizing their importance in addressing antibiotic resistance (Hertweck et al., 2007a, 2007b; Zhang et al., 2017a, 2017b, 2017c, 2017d). NRPS-derived metabolites further contribute to this diversity, especially in the form of cyclic peptides and depsipeptides. Compounds such as thiocoraline and salinosporamide K have demonstrated significant cytotoxic and antitumor activities, highlighting their therapeutic potential. The incorporation of non-proteinogenic amino acids in NRPS products enhances structural complexity and biological specificity (Iacovelli et al., 2021).

**Table 1:** Details of Bioactive compounds from actinomycetes along with the activity and pathway of synthesis.

Actinomycetes	Source	Bioactive compound	NRPS/PKS	Activity	References
*Streptomyces* sp. EGY1	sediment samples obtained from the Red Sea, Egypt.	Sharkquinone (ana-quinonone teracene)	polyketides	induce apoptosis in human gastric adenocarcinoma (AGS) cells	(Elmallah et al., 2020a, 2020b)
*Streptomyces* sp. EGY34	sediment samples obtained from the Red Sea, Egypt.	Resistomycin (pentacyclic polyketide)	polyketides	Cytotoxic in colon HCT116 and breast MDA-MB-231 cancer cell lines And hepatic carcinoma (HepG2) and cervical carcinoma (Hela) cell lines,	(Elmallah et al., 2020a, 2020b)
*Micromonospora chalcea* FIM-02-523	Marine sediment	Rakicidins G-I, Rakicidin E (cyclic Depsipeptides)	PKS/NRPS hybrid	Cytotoxicity against HCT-8 and PANC-1 pancreatic cancer cell lines, effective against gram positive anaerobic bacteria.	(Chen et al., 2018)
*Micromonospora sp.* FIM05328	Marine sediment	Aurodox (Polyene compound)	PKS/NRPS hybrid	Antiproliferative activities against human esophageal squamous tumor cell lines	(Nie et al., 2018a, 2018b)
*Micromonospora sp.* L-13 ACM2-092	Marine coral in Indian Ocean	Thiocoraline (depsipeptide)	NRP	Cytotoxic activities against P388, A549 & SK-MEL28 cell lines, inhibit DNA Polymerase R, antibacterial	(Romero et al., 1997a, 1997b)
*Streptomyces* sp. AP-123	Coast of Bay of Bengal	NA	PKS	Larvicidal activity against *H. armigera* and *S. litura*	(Arasu et al., 2013)
*Nocardiopsis* sp. strain HB-J378	Deep sea	Angucycline (aromatic polyketides)	PKS	against methicillin-resistant *Staphylococcus aureus* (MRSA)	(Xu et al., 2018a, 2018b)
*Streptomyces pratensis* strain NA-ZhouS1	Marine sediment, East China sea	stremycins A and B	PKS	antibacterial activity	Akhter et al. (2018)
*Streptomyces* sp. 182SMLY	Marine sediment	Anthraquinone (anthracenediones)	PKS	Inhibition activity against MRSA	(Liang et al., 2016a, 2016b)
*Micromonospora sp*	Marine brown alga in Bahamas islands	Neaumycin B (polycyclic macrolide)	PKS	cytotoxicity	(Kim et al., 2018a, 2018b)
*Micromonospora sp* 5-297	Marine sediment in Bohai Bay, China	Tetrocarcin N,O (spirotetronate)	PKS	Anti-bacterial	Tan Y et al. (2016)
*Nonomuraea* sp. strain MM565M-173N2	deep-sea sediment, Japan trench	sealutomicins A-D (enediyne)	PKS	Antimicrobial	(Igarashi et al., 2021a, 2021b)
*Streptomyces* sp. EG1	Marine sediment of Mediterranean Sea, Egypt	Mersaquinone (Tetracene)	PKS	inhibited the growth of the methicillin-resistant *S. aureus* (MRSA)	(Kim et al., 2020a, 2020b)
*Streptomyces* sp. HZP-2216E	from a fresh seaweed in South China Sea	bafilomycins (Macrolide)	PKS	growth inhibitory activity against MRSA	(Zhang et al., 2017a, 2017b, 2017c, 2017d)
*Streptomyces althioticus* MSM3	Seaweed in the Cantabrian Sea (Northeast Atlantic Ocean)	desertomycin G (macrocyclic lactone)	PKS	Antibacterial	Brana et al. (2019)
*Micromonospora harpali* SCSIO GJ089	Marine sediment from the northern South China Sea	spirotetronate glycosides, microsporanates A-F	PKS	Inhibitory against MRSA	(Gui et al., 2017a, 2017b)
*Streptomyces koyangensis* SCSIO 5802	Marine sediment from Northern South China Sea	Abyssomicins (polycyclic macrolactones)	PKS	Antibacterial	(Huang et al., 2018a, 2018b)
*Nocardiopsis* sp. NHF48	Marine sediment	Alpha-pyrone	NRPS/PKS Hybrid	Anti- MRSA and activity against mouse melanoma cell line B16	Yang and Song (2018)
*Salinispora pacifica*	Marine sediment	Salinosporamide K	NRPS	Antitumor	Eustaquio et al. (2011)
*Sallinispora arenicola*	Marine sediment	Retimycin A (depsipeptide)	NRPS	Antitumor	(Duncan et al., 2015)
*Salinispora tropica*	Marine sediment	Salinilactam A	PKS	Antibiotic	Udwary et al. (2007)

Hybrid PKS–NRPS pathways play a crucial role in expanding chemical diversity by combining biosynthetic features of both systems. Metabolites such as rakicidins, aurodox, and alpha-pyrone exhibit dual antibacterial and anticancer activities, illustrating the importance of inter-domain coordination in producing multifunctional compounds (Siro and Donald, 2023). The diversity of compounds presented in Table 1 underscores the immense biosynthetic potential of actinomycetes. Their ability to produce structurally unique and biologically active metabolites continues to position them as a cornerstone in natural product-based drug discovery.

## 4 Future prospects

The exploration of actinomycetes and their potential for producing bioactive compounds through NRPS and PKS pathways holds promise for future research and development in the field of pharmaceuticals. The wide distribution of actinomycetes across marine and terrestrial environments provides a rich reservoir for the discovery of structurally diverse and novel bioactive compounds. Further investigations into the physiological adaptations of marine actinomycetes, particularly under extreme environmental conditions, may uncover previously unexpressed biosynthetic pathways and lead to the identification of unique metabolites**.** Advances in understanding the structure and function of NRPS and PKS enzymes, including the communication between domains and modules, may lead to the development of more efficient and effective methods for producing complex molecules. Recent progress in genome mining, synthetic biology, and metabolic engineering offers new opportunities to activate cryptic biosynthetic gene clusters and enhance the production of valuable secondary metabolites. The potential for hybrid PKS-NRPS synthases to create highly complex molecules with unique properties is an exciting area of research. Continued integration of molecular, biochemical, and computational approaches will further facilitate the rational design and discovery of novel compounds with therapeutic relevance.

## 5 Conclusion

Actinomycetes are a promising source of bioactive compounds with pharmaceutical potential, particularly in the areas of NRPS and PKS pathways. Their presence in diverse marine and terrestrial environments, coupled with remarkable physiological adaptability, enables the production of structurally diverse and novel secondary metabolites. The modular organization of NRPS enzymes and the various domains involved in NRP biosynthesis, such as adenylation and thiolation domains, play crucial roles in the effective communication between domains and modules, facilitating the transport of substrates and intermediates. Similarly, PKS enzymes and hybrid PKS–NRPS systems contribute to the biosynthesis of structurally complex molecules with significant biological activities. The extensive diversity of biosynthetic gene clusters in actinomycetes highlights their importance as a reservoir for novel drug discovery. Continued exploration of these pathways, supported by advances in genomics and metabolic engineering, is expected to accelerate the identification and development of new therapeutic agents.

## CRediT authorship contribution statement

Abhishek Chauhan: Conceptualization, Supervision, Writing – review & editing. Manisha Gurnani: Investigation, Data curation, Writing – original draft. Jayati Arora: Methodology, Formal analysis, Writing – original draft. Ritu Chauhan: Resources, Validation, Writing – review & editing. Anuj Ranjan: Visualization, Software, Formal analysis. Laurent Dufossé: Writing – review & editing, Conceptualization. Tanu Jindal: Supervision, Project administration, Funding acquisition.

## AI statement

During the preparation of this manuscript, the authors used AI-assisted tools, including Grammarly and QuillBot, to improve the language and enhance clarity. The authors reviewed and edited the content and take full responsibility for the accuracy, integrity, and originality of the published work.

## Declaration of competing interest

The authors declare that they have no known competing financial interests or personal relationships that could have appeared to influence the work reported in this paper.

## Data Availability

Data will be made available on request.
